# Laparoscopic Resection of Cholecystocolic Fistula and Subtotal Cholecystectomy by Tri-Staple in a Type V Mirizzi Syndrome

**DOI:** 10.1155/2016/6434507

**Published:** 2016-01-24

**Authors:** Fahri Yetişir, Akgün Ebru Şarer, Hasan Zafer Acar, Omer Parlak, Basar Basaran, Omer Yazıcıoğlu

**Affiliations:** ^1^Atatürk Research and Training Hospital, General Surgery Department, Ankara, Turkey; ^2^Atatürk Research and Training Hospital, Anesthesiology and Reanimation Department, Ankara, Turkey; ^3^Natomed Private Hospital, General Surgery Department, Ankara, Turkey

## Abstract

The Mirizzi syndrome (MS) is an impacted stone in the cystic duct or Hartmann's pouch that mechanically obstructs the common bile duct (CBD). We would like to report laparoscopic subtotal cholecystectomy (SC) and resection of cholecystocolic fistula by the help of Tri-Staple*™* in a case with type V MS and cholecystocolic fistula, for first time in the literature. A 24-year-old man was admitted to emergency department with the complaint of abdominal pain, intermittent fever, jaundice, and diarrhea. Two months ago with the same complaint, ERCP was performed. Laparoscopic resection of cholecystocolic fistula and subtotal cholecystectomy were performed by the help of Tri-Staple. At the eight-month follow-up, he was symptom-free with normal liver function tests. In a patient with type V MS and cholecystocolic fistula, laparoscopic resection of cholecystocolic fistula and SC can be performed by using Tri-Staple safely.

## 1. Introduction

Mirizzi syndrome (MS) is a rare cause of intermittent or constant obstructive jaundice, where an impacted stone in the cystic duct or Hartmann's pouch mechanically obstructs the common bile duct (CBD). MS was first described in 1948, as a repeated inflammation of the gallbladder due to an impacted gallstone. This leads to the formation of adhesions between the gallbladder and the CBD resulting in anatomic distortion of these structures [1]. It generally occurs in females with advanced age. Incidence of MS is 0.3–5.7%. Few articles have described the coexistence of MS and cholecystoenteric fistula [2]. Csendes' MS classification system has been renewed. Depending on the degree of involvement of the biliary tract, the patients may be grouped into five distinct groups and coexistence of cholecystoenteric fistula and MS was classified as type V [2]. MS and cholecystoenteric fistulas are rare and late complications of gallstone disease [3, 4]. The most common type of biliary enteric fistula is cholecystoduodenal (75%); cholecystocolic fistula is the next common (10–20%), with a variety of other types being less common (15%) [5]. Symptoms are similar to that of acute and chronic cholecystitis, with or without jaundice and diarrhea [1].

It is important to identify MS and fistula formation before surgery or at least during surgery because of serious morbidity and mortality related to the condition [3]. Correct surgical approach and management are very important for MS as chronic biliary tree inflammation and bile duct anatomic alteration necessitate a rigorous technique [5].

Laparoscopic subtotal cholecystectomy (SC) is considered to be a safe option in severe cholecystitis with frozen anatomy within Calot's triangle where there is a potential risk of causing injury to the common bile duct (CBD) [6]. Laparoscopic stapling devices are an effective method for performing LSC or dividing a dilated cystic duct or neck of gallbladder due to its ease and speed of application [7].

According to our knowledge there is no reported data about laparoscopic treatment of type V MS. We would like to report laparoscopic SC and resection of cholecystocolic fistula by the help of endoscopic stapler (Covidien Endo GIA Reinforced Reload with Tri-Staple Technology) in a case with type V MS with cholecystocolic fistula.

## 2. Presentation of Case

A 24-year-old man was admitted to emergency department with the complaint of abdominal pain, intermittent fever, jaundice, and diarrhea for the last five days. Pain especially was present at right upper quadrant and epigastric region. In his past history, there was no operation and chronic disease. He had been admitted 2 months ago with the same complaint and hospitalized for cholelithiasis and choledocolithiasis. Endoscopic retrograde cholangiopancreatography (ERCP) was performed. Endoscopic papillotomy was applied and sludge was removed by ERCP. Five days later, total bilirubin level decreased from 3.1 to 1.2 mg/dL (normal 0.2–1 mg/dL), and the patient was discharged from gastroenterology department. His vital parameters were as follows: blood pressure (BP): 110/50 mmHg, heart rate (HR): 88, and fever: 37.7°C. On abdominal examination, there was evidence of icterus and sensitiveness and rebound was positive at right hypochondrium. In biochemical analysis, total bilirubin was 2.6 mg/dL, conjugated bilirubin 1.8 (normal < 0.2 mg/dL), serum glutamic oxaloacetic transaminase (SGOT) 60 U/L (5–40 U/L), serum glutamic-pyruvic transaminase (SGPT) 71 U/L (normal 5–40 U/L), alkaline phosphatase 287 U/L (<106 U/L), and glutamyl transferase 130 U/L (<45 U/L). LDH was 277 U/L, lipase 155 U/L, amylase 134 U/L, and CRP 7 mg/dL; in total blood count, WBC was 12.000 K/Ul. Abdominal ultrasound showed that so many calculi were present in gallbladder and a large calculus was located in cystic duct. He underwent emergent laparoscopic operation. The final diagnosis was made intraoperatively.

### 2.1. Surgical Technique

**Figure 1:**
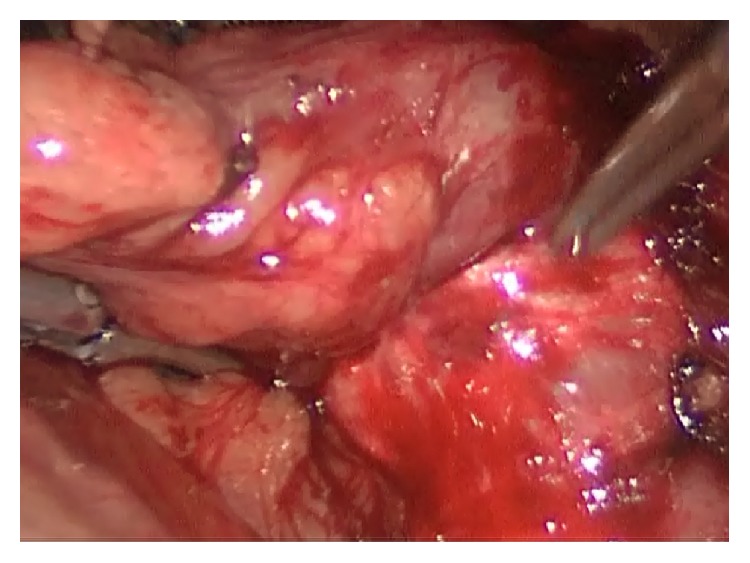
Cholecystocolic fistula between fundus of gallbladder and hepatic flexure of colon is seen.

**Figure 2:**
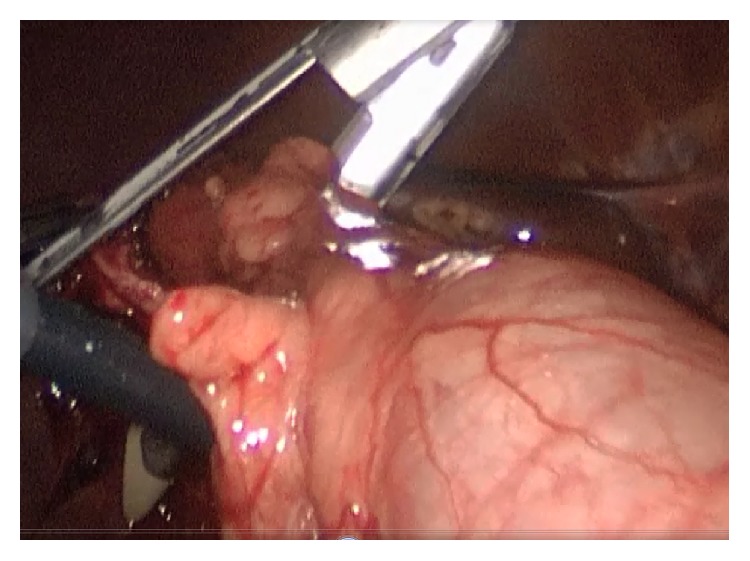
Resection of the cholecystocolic fistula by the help of the vascular Tri-Staple is seen (Covidien Endo GIA Reinforced Reload with Tri-Staple Technology).

**Figure 3:**
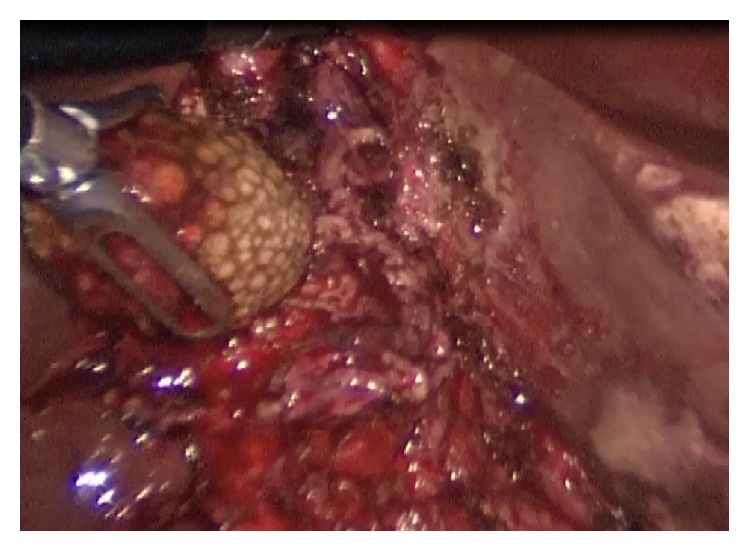
Extraction of large impacted stone from cholecystocholedochal fistula trough opening of fundus is seen.

**Figure 4:**
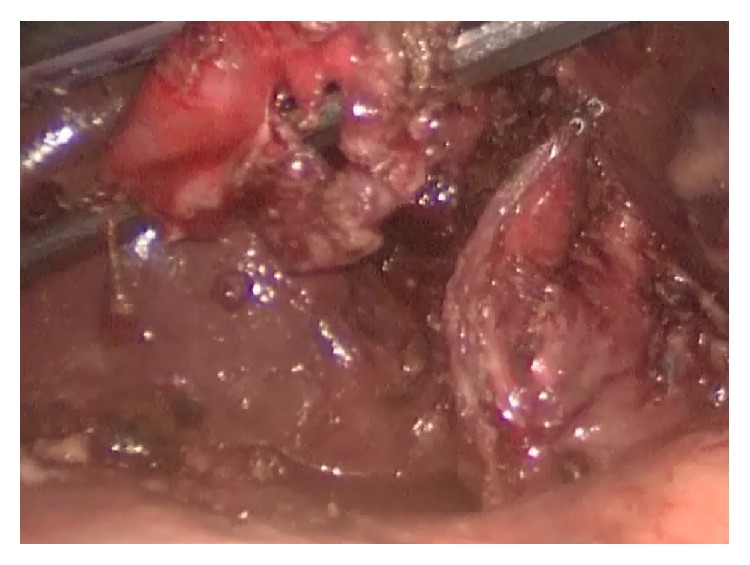
Applied Tri-Staple line is seen on stump.

**Figure 5:**
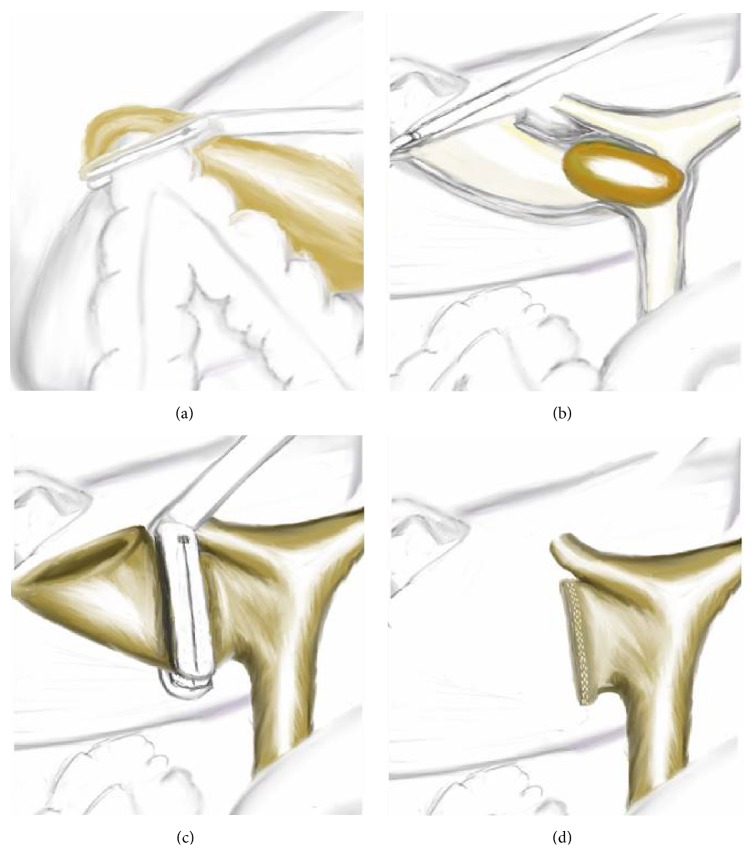
Schematic illustration of the case: (a) illustration is seen during the resection of cholecystocolic fistula by Tri-Staple. (b) Impacted stone is seen in the cholecystocholedochal fistula like type IV Mirizzi syndrome, illustration is drown as anterior surface of gallbladder and biliary was opened. (c) Subtotal cholecystectomy line by Tri-Staple is seen. (d) After completion of the subtotal cholecystectomy, stump of gallbladder and staple line on stump is seen.

He underwent operation under general anesthesia. Classical 4 trocars entries were used. There were dense adhesions between colon, duodenum, gallbladder, and omentum in the right subhepatic space. After gentle dissection, first cholecystocolic fistula between fundus of gallbladder and hepatic flexure of colon was observed (Figure 1). After visualization of boundaries of cholecystocolic fistula, its resection was performed by the help of the vascular Tri-Staple (Covidien Endo GIA*™* Reinforced Reload with Tri-Staple Technology) (Figure 2). It was seen that there was no narrowing at resection side of colon. Later there were significant difficulties in dissecting the gallbladder neck and Calot's triangle and further dissection would expose the patient to a higher risk of common bile duct injury or hemorrhage; the cystic duct and artery could not be isolated. The operative method was changed and dissection was started from fundus and anterior wall of fundus was resected. All gallstones were extracted one by one through an incision in the fundus of gallbladder. At the end large impacted stone was also extracted (Figure 3). After extracting this large impacted stone, excessive amount of bile drainage came from this fistula. Irrigation of common bile duct with saline was performed via fistula opening. There were no other stones. A subtotal cholecystectomy was done by stapling over infundibulum of gallbladder with medium thick Tri-Staple (Figure 4). All the resection steps were summarized in illustration (Figure 5). Resection of the remnant posterior part of fundus could be completed by harmonic scalpel. After irrigation with saline, one drainage was placed near to the gallbladder stump. Postoperative course was uneventful and patient was discharged 5 days after the operation. At the eight-month follow-up, there was no problem. He was symptom-free with normal liver function tests.

## 3. Discussion

During laparoscopic treatment of this case, two important problems were facilitated by using laparoscopic stapler; one of them is resection of cholecystocolic fistula and second one is subtotal cholecystectomy. By the help of stapler resection of cholecystocolic fistula was performed laparoscopically without injuring or narrowing the colon. After extracting impacted stone and irrigating CBD with saline subtotal cholecystectomy was undertaken by applying a staple across gallbladder infundibulum safely. Before subtotal cholecystectomy to understand anatomical structure of external bile duct using cholangiogram or choledochoscopy could be better but we could not perform both of them due to technical insufficiencies. Suturing the gallbladder stump would be an acceptable alternative if stapler could not be applied.

Open surgery for MS is accepted. The reported conversion rate to open cholecystectomy was remarkably high, with a range of 37–78% [8]. As the type of MS increases, conversion rate increases with concordance. There is no reported data in literature about laparoscopic treatment of a case with type V MS and cholecystocolic fistula.

There are so many treatment modalities for MS. It can be changed according to patient, type of MS, and experience of surgeon and gastroenterologist to apply ERCP. If the patient has high risk for surgery, endoscopic treatment can also be tried [9]. Laparoscopic or open SC can be used safely by dividing across Hartmann's pouch, infundibulum, or dilated cystic duct. Another safe option is the drainage of the remnant pouch after performing a partial cholecystectomy. The proper surgical treatment mentioned in literature is partial cholecystectomy and a biliary drainage procedure (Roux-en-Y anastomosis), with open surgical procedure especially in types III and IV MS [10].

There are two most important issues for SC; one of them is the formation of residual gallstones in the remnant gallbladder. Symptomatic gallstone disease recurrence was reported to be 2.2–5%. These complications can be treated successfully by endoscopic papillotomy or completion with cholecystectomy. Another one is gallbladder cancer which is found in 0.2–0.8% of patients undergoing laparoscopic cholecystectomy [10, 11].

Chaudery et al. reported two laparoscopic subtotal cholecystectomies by using stapler and both of them had acute pancreatitis approximately 6 weeks after this operation. The episodes of pancreatitis described in their cases were thought to be associated with contained residual stones in the remnant pouch. At the end they advised that laparoscopic SC with stapler should be done after extracting all stones and irrigating CBD via the opening of the fundus [7]. In our case no complication occurred, because first all the stone was removed and irrigation of CBD was done; after that reticulating endoscopic stapler was applied to close infundibulum of gallbladder in a good exposure by using 30°C scope. Another factor may be the application of the endoscopic papillotomy by ERCP before surgery for preventing from this kind of complication.

## 4. Conclusion

Laparoscopic resection of cholecystocolic fistula and subtotal cholecystectomy can be performed by using Tri-Staple safely in a patient with type V MS and cholecystocolic fistula.
